# Curative GnRHa treatment has an unexpected repressive effect on Sertoli cell specific genes

**DOI:** 10.1186/s12610-018-0067-1

**Published:** 2018-02-09

**Authors:** Katharina Gegenschatz-Schmid, Gilvydas Verkauskas, Philippe Demougin, Vytautas Bilius, Darius Dasevicius, Michael B. Stadler, Faruk Hadziselimovic

**Affiliations:** 1 Cryptorchidism Research Institute, Kindermedizinisches Zentrum Liestal, 4410 Liestal, Switzerland; 2 Children’s Surgery Centre, Faculty of Medicine, Vilnius University, 01513 Vilnius, Lithuania; 3 Biozentrum, Life Sciences Training Facility, University of Basel, 4001 Basel, Switzerland; 4 Institute for Pathology, National Centre of Pathology, Affiliate of Vilnius University Hospital Santariskiu Klinikos, 08406 Vilnius, Lithuania; 5 Friedrich Miescher Institute for Biomedical Research, 4058 Basel, Switzerland; 6 Swiss Institute of Bioinformatics, Basel, Switzerland

**Keywords:** Sertoli cells, Ad spermatogonia, RNA-Sequencing, LH, Testosterone, GnRHa-treatment, Infertility, Cryptorchidism, Mini-puberty, Cellules de Sertoli, Spermatogonies Ad, Séquençage des ARN, LH, Testostérone, Traitement par GnRHa, Infécondité, Cryptorchidie, Minipuberté

## Abstract

**Background:**

Follicle stimulating hormone and testosterone stimulate Sertoli cells to support germ cell function and differentiation. During mini-puberty, when gonadotropin (GnRH) stimulates increases in plasma luteinizing hormone (LH) and testosterone levels, gonocytes are transformed into Ad spermatogonia. In cryptorchidism, impaired gonadotropin secretion during mini-puberty results in insufficient LH and testosterone secretion, impaired gonocyte transition to Ad spermatogonia, and perturbed Sertoli cell proliferation. Treatment with a gonadotropin-releasing hormone agonist (GnRHa/Buserelin) induced gonocytes to differentiate into Ad spermatogonia and rescued fertility. The present study evaluated the impact of low LH secretion on Sertoli cell function by comparing differential gene expression data between testes with low LH that lacked Ad spermatogonia (Ad-) and testes that completed mini-puberty (Ad+). Furthermore, we analyzed changes in the transcription of selected Sertoli cell specific genes in response to GnRHa treatment.

**Results:**

Ad- testes showed reduced expression of nine out of 40 selected Sertoli cell specific genes compared to Ad+ testes. GnRHa treatment repressed most of the Sertoli cell specific genes, including the inhibins, but it increased the expression of genes that regulate apoptosis (*FASLG*) and proliferation (*GDNF*).

**Conclusions:**

Impaired-minipuberty with decreased LH and testosterone levels affected Ad and Sertoli cell development through positive and negative regulation of morphoregulatory and apoptotic genes. GnRHa treatment had a repressive effect on most Sertoli cell specific genes, which suggested that Sertoli cells underwent a cellular rearrangement. We propose that gonadotropin-dependent increases in *FASLG* and *GDNF* expression drove Sertoli cell proliferation and germ cell self-renewal and supported the transition of gonocytes to Ad spermatogonia, independent of inhibins.

## Résumé

**Contexte:**

L’hormone folliculostimulante et la testostérone stimulent les cellules de Sertoli pour soutenir la fonction et la différentiation des cellules germinales. Pendant la minipuberté, lorsque la gonadotrophine (GnRH) stimule les augmentations des taux plasmatiques d’hormone lutéinisante (LH) et de testostérone, les gonocytes sont transformés en spermatogonies Ad. Dans la cryptorchidie, une sécrétion altérée de gonadotrophine lors de la minipuberté entraine une sécrétion insuffisante de LH et de testostérone, une altération de la transition des gonocytes en spermatogonies Ad, et une perturbation de la prolifération des cellules de Sertoli. Un traitement par agoniste de la gonadolibérine (GnRHa/Buserelin) induit une différenciation des gonocytes en spermatogonies Ad et sauvegarde la fertilité.

Cette étude a évalué l’impact d’un taux de LH bas sur la fonction des cellules de Sertoli en comparant les données d’expression différentielle de gènes entre des testicules avec LH basse qui sont dépourvus de spermatogonies Ad (Ad-) et des testicules qui ont fini la minipuberté (Ad+). En outre, la réponse au traitement par GnRHa a été analysée par les modifications de la transcription de gènes sélectionnés pour être spécifiques de la cellule de Sertoli.

**Résultats:**

Les testicules Ad- présentent une expression réduite de 9 des 40 gènes sélectionnés pour être spécifiques de la cellule de Sertoli par comparaison aux testicules Ad+. Le traitement par GnRHa a réprimé l’expression de la plupart des gènes spécifiques de la cellule de Sertoli, y compris les inhibines, mais a augmenté l’expression de gènes qui régulent l’apoptose (FASLG) et la prolifération (GDNF).

**Conclusions:**

Une minipuberté altérée par des taux diminués de LH et de testostérone affecte le développement des spermatogonies Ad et des cellules de Sertoli par une régulation positive et négative de gènes morphorégulateurs et apoptotiques. Le traitement par GnRHa a un effet inhibiteur sur la plupart des gènes spécifiques de la cellule de Sertoli, ce qui suggère que les cellules de Sertoli subissent un réarrangement cellulaire. Nous proposons que les augmentations gonadotrophine-dépendantes de l’expression de FASLG et de GDNF dirigent la prolifération des cellules de Sertoli et l’auto-renouvèlement des cellules germinales, et qu’elles sont le support de la transition gonocytes vers spermatogonies Ad, indépendante des inhibines.

## Background

During prepuberty four types of Sertoli cells develop under hormonal control [1]: fetal Sertoli cells (Sf), which are observed in the first months after birth; the Sa and Sb types, which are observed during childhood; and the Sc type, which is observed in puberty and throughout life. In the first three months after birth, activation of the hypothalamic-pituitary-gonadal (HPG) axis leads to a transient increase in gonadotropins and testosterone [2–4], termed mini-puberty. During mini-puberty, the number of Sertoli cells increases [5, 6].

Within the seminiferous tubules, Sertoli cells possess receptors for testosterone and follicle stimulating hormone (FSH). Gonadotropin releasing hormone (GnRH) drives testosterone and FSH signaling. In Sertoli cells, this signaling promotes the production of factors that support germ cell function and differentiation. Sertoli cells secrete anti-Müllerian hormone (AMH), activins, inhibins, glial cell-derived neurotrophic factor (GDNF), KIT ligand (KITLG), and FAS ligand (FASLG). Prepubertal Sertoli cells express subunits of the active inhibin B [7], which counteracts the effect of activin A by suppressing FSH secretion in gonadotropes [8, 9].

The testosterone-dependent transition of gonocytes into A dark (Ad) spermatogonia during mini-puberty is an essential process for fertility [10, 11]. Prepubertal human testes with defective mini-puberty lacked the transition of gonocytes into Ad spermatogonia (Ad-) and showed a diminished number of Sf and Sb Sertoli cells [12, 13]. Those findings were consistent with recent observations of significantly reduced plasma levels of testosterone, AMH, and inhibin B in boys with cryptorchidism [14]. The early postnatal increase in inhibin B during mini-puberty [15] is presumably due to the activation of the HPG axis [16], and it reflects the proliferation of Sertoli cells. Of interest, the early postnatal rise in inhibin B was correlated more closely with luteinizing hormone (LH) and testosterone levels than with FSH levels [17, 18]. That finding raised the possibility that Sertoli cell proliferation in neonatal life might depend more on LH/testosterone than on FSH. Studies in the literature have reported equivocal findings of normal, decreased, and increased inhibin B secretion in boys with cryptorchidism [9, 19–24]. Cortes and colleagues found a positive correlation between inhibin B and Ad spermatogonia by analyzing 2-μm thick sections of paraffin embedded specimens [25]. However, a more precise analysis method that employed 1-μm semi-thin sections of Epon embedded specimens could not confirm the earlier report of a correlation between the number of Ad spermatogonia and plasma inhibin B levels [26].

Treatment with the gonadotropin-releasing hormone agonist (GnRHa), Buserelin, induced an increase in LH and testosterone [27–29] and rescued fertility in the majority of pathological cryptorchid testes. Buserelin induced expression of genes in the HPG axis [30] and genes involved in gonocyte transition to Ad spermatogonia [31]. Ultrastructural changes within Sertoli cells after GnRHa treatment included the presence of abundant lipid droplets, changes in nuclear form, and increased amounts of rough endoplasmic reticulum in the cytoplasm [27]. In a randomized double-blind, placebo-controlled study cryptorchid boys were treated with a low dose (20 micrograms) of GnRHa, given as a nasal spray, for a short period of 28 days. Boys treated with Buserelin had the highest number and the best maturation index of germ cells [32]. Furthermore, in 2007 it has been shown that patients treated with Buserelin and with a normal Leydig cell testosterone secretory capacity will have normal testicular histology and Ad spermatogonia. Those with a suboptimal Leydig cell testosterone secretory capacity will have a low Ad spermatogonia count and consequently poor prognosis for future fertility, despite successful surgery [29].

In this study, we analyzed the impact of low (hypogonadotropic) LH on differential expression of specific Sertoli cell genes between Ad- testes with impaired mini-puberty and Ad+ testes that completed mini-puberty. Furthermore, we studied the effect of GnRHa on Sertoli cells by analyzing differential Sertoli cell specific gene expression after GnRHa treatment in Ad- testes. Our findings extended our understanding of how Sertoli cells and their secreted factors, including inhibins, are involved in the gonocyte transition to Ad spermatogonia.

## Methods

### Study population and biopsy sample collection

We selected 15 patients with isolated cryptorchidism, based on histological results, and divided them into 2 groups. Seven belonged to the Ad− (lacking Ad spermatogonia) and 8 to the Ad+ (presenting Ad spermatogonia) group. The patients had a median age of 18.5 months (range 8–59 months). Data from Ad− bilateral cryptorchid boys treated with GnRHa (Buserelin) following the first orchidopexy (surgery) (4 patients) were retrieved from an ongoing randomized study. Initial biopsies revealed no Ad spermatogonia, indicating defective mini-puberty (Ad− group). The second testis was managed by orchidopexy and biopsied 6 months after the initial surgery. Thus, results from 21 biopsies were compared. Patients were age and ethnicity matched. RNA sequencing data from our two previous studies [30, 33] were used to analyze manually selected genes expressed specifically in Sertoli cells.

A cryptorchid testis is defined as a testis localized outside of the scrotum and incapable of being brought into a stable scrotal position. All undescended testes in this study were located in the inguinal region. Testicular biopsies were taken at the time of orchidopexy. This sample was then subdivided, with one fragment fixed in glutaraldehyde for histological processing, while the other one was immediately immersed in RNAlater (ThermoFisher Scientific, Waltham, Massachusetts, USA) and stored at –25 °C until further processing (for RNA extraction and RNA- sequencing).

### Histological analyses

Biopsies were fixed in 3% glutaraldehyde in phosphate-buffered saline (PBS, pH 7.4) and then embedded in Epon resin. Semi-thin sections (1 μm) were cut using a Reichert Om-U3 ultramicrotome (Reichert AG, Vienna, Austria). Sections were mounted on glass slides, stained with 1% toluidine blue, and examined under a Zeiss Axioskop light microscope (Carl Zeiss Microscopy GmbH, Jena, Germany) with an integrated photo-camera. Biopsies were histologically examined by two of the authors (F.H. and D.D.), each with expertise in the interpretation of semi-thin sections of prepubertal testes.

During histological analyses, at least 100 tubular cross sections per biopsy were evaluated, with regard to their number of spermatogonia per tubule (S/T) and presence of Ad spermatogonia. In the prepubertal testes, Ad spermatogonia were identified according to the criteria first published by Seguchi and Hadziselimovic [34]. This type of germ cell has a typical halo in the nucleus, termed the rarefaction zone, and cytoplasm with a darker aspect in comparison to Ap or fetal spermatogonia. For each biopsy, at least 100 tubular cross sections were evaluated. On the basis of this evaluation, biopsies were categorized into 2 groups, Ad- (high infertility risk, HIR) and Ad+ (low infertility risk, LIR), dependent on their infertility outcome. The Ad- group included biopsies with S/T ≤0.2 and no Ad and the Ad+ group recorded S/T scores of >0.6 with Ad. Cryptorchid boys in the Ad- group had 8 times lower plasma LH (0.11 IU/L) compared to the Ad+ group (0.89 IU/L, *p*<0.009) indicating hypogonadotropic hypogonadism [26].

### RNA preparation, sequencing, data analyses, and RNA expression levels

The workflow from RNA isolation, through to purification, library preparation, sequencing, data analyses, and expression level analysis, has been previously described in detail [30, 33].

### Data and differential gene expression analyses

Determination of differentially expressed genes, statistical analyses and model design were described previously [30, 33]. Only genes with at least one read per million, in at least two samples, were included. *P* values and fold-changes were calculated for the treatment factor and differentially expressed genes were defined as those displaying a false discovery rate (FDR) of less than 0.05. Raw data files are available at the Database of Genotypes and Phenotypes (dbGaP) with the accession number phs001275.v1.p1.

## Results

We analyzed the expression data of 40 manually selected Sertoli cell expressing genes [35], whose expression or protein products are commonly used to identify or differentiate Sertoli cells from other testicular cells at various developmental stages. The results were described as fold change (FC) of gene expression, log 2-fold-change (LogFC) and false discovery rate (FDR) between the tested groups (Ad- vs. Ad+ and GnRHa treated vs. untreated) in Table 1. A positive LogFC indicated a gene upregulation and a negative LogFC a gene downregulation in Ad- testes relative to Ad+ testes or in GnRHa treated versus untreated testes (Table 1).

**Table 1:** Differential expression of Sertoli cell specific genes in Ad- versus Ad+ group (Ad-/Ad+) and in the GnRHa treated versus untreated group (GnRHa)

Gene ID	Name	FC Ad-/Ad+	logFC Ad-/Ad+	FDR Ad-/Ad+	FC GnRHa	logFC GnRHa	FDR GnRHa
*AMH*	Anti-Mullerian Hormone	-	n.s.	n.s.	-	n.s.	n.s.
*AR*	Androgen Receptor	-	n.s.	n.s.	*1.5*	-0.5821	0.0126
*BMP4*	Bone Morphogenetic Protein 4	-	n.s.	n.s.	-	n.s.	n.s.
*BMP6*	Bone Morphogenetic Protein 6	*1.7*	-0.7612	0.0030	-	n.s.	n.s.
*CALB2*	Calbindin 2	-	n.s.	n.s.	2.2	-1.1556	0.0034
*CDKN1B*	Cyclin-Dependent Kinase inhibitor 1B (p27, Kip1)	-	n.s.	n.s.	*1.7*	-0.7845	0.0016
*CLDN11*	Claudin 11	-	n.s.	n.s.	*1.8*	-0.8315	0.0011
*CLU*	Clusterin	-	n.s.	n.s.	*1.9*	-0.9548	0.0006
*CREB1*	cAMP Responsive Element Binding protein 1	*1.1*	0.2000	0.0489	*1.6*	-0.6888	0.0041
*CTSL*	Cathepsin L	-	n.s.	n.s.	*1.7*	-0.7492	0.0020
*DES*	Desmin	2.8	-1.4882	0.0014	-	n.s.	n.s.
*DHH*	Desert Hedgehog	-	n.s.	n.s.	-	n.s.	n.s.
*DMRT1*	Doublesex and Mab-3 Related Transcription factor 1	-	n.s.	n.s.	*1.7*	-0.7838	0.0010
*FAS*	Fas cell surface death receptor	-	n.s.	n.s.	-	n.s.	n.s.
*FASLG*	Fas Ligand (TNF superfamily, member 6)	-	n.d.	n.d.	5.0	2.3154	0.0031
*FSHR*	Follicle stimulating hormone receptor	*1.4*	0.4915	0.0363	-	n.s.	n.s.
*FGF2*	Fibroblast Growth Factor 2	-	n.s.	n.s.	-	n.s.	n.s.
*FGF9*	Fibroblast Growth Factor 9	2.1	-1.0605	0.0016	-	n.s.	n.s.
*GATA1*	GATA binding protein 1 (globin transcription factor 1)	-	n.d.	n.d.	-	n.d.	n.d.
*GATA4*	GATA binding protein 4	-	n.s.	n.s.	-	n.s.	n.s.
*GJA1/CX43*	Gap Junction protein, Alpha 1/Connexin 43	*1.3*	0.4018	0.0324	*1.9*	-0.9516	0.0002
*GDNF*	Glial cell Derived Neurotrophic Factor	-	n.s.	n.s.	2.8	1.4687	0.0036
*INHA*	Inhibin, alpha	-	n.s.	n.s.	*1.8*	-0.8158	0.0023
*INHBA*	Inhibin, Beta A	-	n.s.	n.s.	*1.6*	-0.6363	0.0124
*INHBB*	Inhibin, Beta B	-	n.s.	n.s.	*1.6*	-0.6616	0.0058
*KATNAL1*	Katanin p60 subunit A-like 1	-	n.s.	n.s.	*1.7*	-0.7782	0.0012
*KITLG*	KIT Ligand	-	n.s.	n.s.	2.1	-1.0995	0.0001
*KRT18*	Keratin 18, type I	-	n.s.	n.s.	*1.5*	-0.5730	0.0176
*NR5A1/SF1*	Nuclear Receptor subfamily 5, group A, member 1	-	n.s.	n.s.	-	n.s.	n.s.
*PTGDS*	Prostaglandin D2 Synthase	-	n.s.	n.s.	-	n.s.	n.s.
*SERPINA5*	Serpin Family A Member 5	-	n.s.	n.s.	*1.7*	-0.7511	0.0076
*SOX8*	SRY (sex determining region Y)-box 8	2.8	-1.4641	0.0003	-	n.s.	n.s.
*SOX9*	SRY (sex determining region Y)-box 9	*1.4*	0.4983	0.0300	-	n.s.	n.s.
*SRY*	Sex determining Region Y	-	n.s.	n.s.	-	n.s.	n.s.
*TF*	Transferrin	-	n.s.	n.s.	-	n.s.	n.s.
*TJP1/ZO1*	Tight Junction Protein 1/Zona Occludens 1	-	n.s.	n.s.	*1.6*	-0.6824	0.0102
*VIM*	Vimentin	-	n.s.	n.s.	*1.5*	-0.5901	0.0352
*WT1*	Wilms tumor 1	-	n.s.	n.s.	*1.5*	-0.5654	0.0180
*ZFPM1/FOG1*	Zinc Finger Protein, FOG family member 2	-	n.s.	n.s.	-	n.s.	n.s.
*ZFPM2/FOG2*	Zinc Finger Protein, FOG family member 2	*1.3*	0.3695	0.0240	-	n.s.	n.s.

Absolute fold change (FC), Log 2-fold change (logFC), false discovery rate (FDR), not significant (n.s.), not determined (n.d.)

Absolute fold changes (FC) <2 are highlighted in italic

### Nine of 40 Sertoli cell specific genes were differentially expressed in Ad- testes

**Fig. 1:**
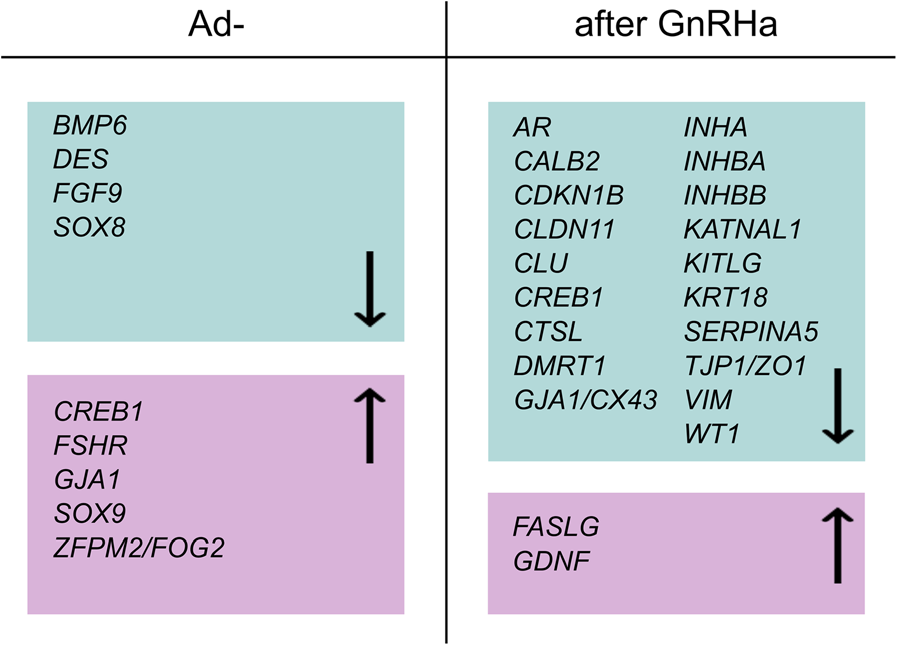
Graphical abstract of up- and downregulated genes in Ad- compared to Ad+ testes and after GnRHa treatment

We found four genes that showed lower expression in Ad- testes compared to Ad+ testes: *BMP6* with 1.7 fold-, *DES* with 2.8 fold-, *FGF9* with 2.1 fold-, and *SOX8* with 2.8 fold decreased expression (Fig. 1 and Table 1). The differential expression values of the genes *BMP6* [30], *FGF9* [33], and *SOX8* [30] between the Ad- and Ad+ group was described by us earlier.

Five genes (*GJA1*, *CREB1*, *FSHR*, *ZFPM2*/*FOG2*, and *SOX9* (described by us earlier [30])) displayed increased RNA levels in Ad- testes compared to Ad+ testes (Fig. 1 and Table 1).

We noted that 29 Sertoli cell specific genes were not significantly differentially expressed. Moreover, the expression levels of *FASLG* and *GATA1* were not detectably different between the two groups.

### GnRHa affects Sertoli cell specific genes

We found that GnRHa treatment had mostly a negative effect (48%) by reducing expression profiles of Sertoli cell specific genes. GnRHa treatment downregulated 19 Sertoli cell specific genes (*AR*, *CLU*, *INHA*, *INHBA*, *INHBB*, *KRT18*, *VIM*, *KATNAL1*, *GJA1*, *TJP1*, *CLDN11*, *SERPINA5*, *CDKN1B*, *CALB2*, *CTSL*, *CREB1*, *DMRT1* [31], *WT1*, and *KITLG* [31]) (Fig. 1 and Table 1). In an earlier study, we reported that GnRHa treatment had a negative effect on the gene expression of secreted Sertoli cell factor, *KITLG*, and the transcription factor, *DMRT1*, which are essential during germ cell development and differentiation [31]. Interestingly, only two genes (*CREB1* and *GJA1*) were differentially expressed between Ad- and Ad+ testes.

We found that GnRHa treatment highly upregulated the gene expression of *FASLG* (fivefold) and *GDNF* (2.8 fold). The strongly upregulated RNA expression of *GDNF* after GnRHa treatment was described by us earlier [31]. Eighteen genes did not show significant differential RNA expressions, and *GATA1* expression was not detected.

## Discussion

### Positive and negative regulators mediate LH-dependent Sertoli cell development

Contrary to our expectation, only nine out of 40 Sertoli cell specific genes were differentially expressed in LH-deficient Ad- testes compared to Ad+ testes (Table 1). Inhibin B, a dimer of α and β subunits (*INHA* and *INHBB*), is mainly produced by Sertoli cells; it negatively regulates the release of pituitary FSH and antagonizes the stimulating action of activin A [36–39]. Of importance, no differential expression was observed in the single inhibin subunits, *INHA*, inhibin βA (*INHBA*), and *INHBB*, between the two studied groups. This finding supported the observation that plasma values for inhibin B were similar between Ad- and Ad+ testes [26]; moreover, this finding brings into question the hypothesis that inhibin B is essential for the transformation of gonocytes into Ad spermatogonia [25]. Alternatively, we propose that, during mini-puberty, LH and testosterone, but not inhibin B, drive the differentiation of gonocytes into Ad spermatogonia.

Bone morphogenetic protein 6 (BMP6) was reported to inhibit apoptosis and to influence the production of tight junction protein 1 (TJP1), GDNF, and KITLG in Sertoli cells [40]. Although we observed a downregulation of *BMP6*, the expression levels of *TJP1*, *KITLG*, and *GDNF* were not significantly (FDR>0.05) altered between the two groups.

Fibroblast growth factor 9 (FGF9) is a downstream effector of GATA4, ZFPM2, SRY, and SOX9 signaling pathway during male sex determination. FGF9 regulates Sertoli cell differentiation through FGFR2 signaling [41, 42]. Both transcription factors, SOX8 and SOX9, can induce AMH expression, and it was suggested that these transcription factors might be redundant in testis differentiation [43, 44]. In Ad- testes, *FGF9* and *SOX8* expression levels were reduced more than two-fold, but *ZFPM2* and *SOX9* were slightly increased (1.3 fold and 1.4 fold, respectively) (Table 1, [30]); this result suggested that function of Sertoli cells was impaired in Ad- testes.

The cAMP response element binding protein 1 (CREB1) is a member of the family of transcription factors that are responsive to hormones, which are critical in nearly all mammalian cell types for development and differentiation. FSH [45] and testosterone (non-classical) activate signaling pathways [46] that lead to phosphorylation/activation of CREB1 in the nucleus of Sertoli cells. Similarly, GDNF-signaling leads to phosphorylation of CREB1 in germ cells [47]. We observed 1.1 fold increased *CREB1* RNA expression in Ad- testes; however, we did not evaluate the activation state of CREB1 protein.

Alterations in gap junction protein alpha 1 (*GJA1*, also known as *Connexin 43*) expression were previously associated with different forms of spermatogenic impairment in men [48–54]. Furthermore, GJA1-based gap junctions have been reported to form a transverse and longitudinal intercellular communication network within seminiferous tubules, which ensures the synchronization of germ cell proliferation and differentiation [55]. However, the effect of an absolute fold change of 1.3 in *GJA1* expression in Ad- testes remains unexplained.

### GnRHa-treatment suppresses gene expression in Sertoli cells

GnRHa treatment caused downregulation of the cytoskeleton-related genes, *KRT18*, *VIM*, and *KATNAL1*, and the cell junction protein encoding genes, *GJA1*, *TJP1*, and *CLDN11*. This downregulation pointed to a structural remodeling process, both within Sertoli cells and in cell-cell connections between individual Sertoli cells and between Sertoli cells and germ cells.

Clusterin (CLU) was originally identified as an androgen/testosterone-repressed gene in the prostate [56] and reduced CLU protein levels in testis have been linked to male infertility [57, 58]. In a recent report, it was demonstrated that CLU conferred protection against apoptosis and appeared to induce meiosis in male germ cells via VLDLR/LPR8 receptors in rats [59]. GnRHa treatment suppressed the expression of both these receptors (VLDLR logFC^GnRHa^ -0.74 and LPR8 logFC^GnRHa^ -0.76). We suggest that the observed reduced levels of androgen-sensitive *CLU* expression resulted from GnRHa-induced increases in testosterone, and that this activity inhibited germ cells from entering premature meiosis.

Furthermore, we observed reductions in RNA expression of *CDKN1B*, *CALB2*, *CTSL*, *CREB1*, *DMRT1* [31], and *WT1*. All these genes are involved in Sertoli cell function and differentiation. This finding suggested that GnRHa induced transcriptional changes in multiple target genes.

Cells that express AR in human testis include Sertoli cells, Leydig cells, peritubular myoid cells, arteriole smooth muscle cells, and vascular endothelial cells. Immunostaining methods for detecting AR protein showed rather poor expression in human prepubertal Sertoli cells, but AR expression increased after the age of four years [60–63]. Furthermore, *AR* expression is modulated by testosterone and FSH [64, 65]. Therefore, the observed reduction in *AR* expression could be due to a GnRHa-dependent increase in testosterone in treated testes. Analyzing ARKO mice, Yue and coworkers compared expression levels of *Ddx4*, *Amh*, *c-Kit*, *Mmp14*/*Mt1-mmp*, *Rhox5*, *Zbtb16*, and *Pou5f1* genes. They concluded that gonocyte transformation to A-type spermatogonia was androgen-independent in mouse and human. However, they did not analyze genes specific for Ad spermatogonia, like *T*/*BRACHYURY*, *TERT*, *PAX7*, *DMRTC2*, and *FGFR3*. Furthermore, Li et al. performed immunofluorescence histochemistry to detect the proliferation marker, Ki67, the Sertoli cell marker, AMH, and the germ-cell marker, DDX4. They did not observe any differences in gonocyte migration from the center to the tubular basement membrane, or in spermatogonial stem cell transformation [66]. Thus, they concluded that gonocyte transition to Ad spermatogonia and gonocyte migration towards the basement membrane were androgen-independent processes in both mouse and human. Although the expression of spermatogonial markers is conserved in species ranging from rodents to primates, species differences have been reported, including the succession of markers and their correlations with the differentiation state of spermatogonia (reviewed [67, 68]). This species discrepancy was true for DDX4; in human, DDX4 was not localized in gonocytes, but its onset of expression was associated with the formation of spermatogonia [69]. Notably, there are different types of spermatogonia [34]. It is of paramount importance to realize that all spermatogonia are DDX4-positive cells, including spermatogonial stem cells (SSCs). In contrast to the findings of Li and co-workers, our results led us to conclude that, in infants with cryptorchidism and defective mini-puberty, testosterone is essential for the gonocyte-to-Ad spermatogonia transition; moreover, we concluded that testosterone had an effect after GnRHa treatment on expression of Sertoli cell specific genes, such as *AR*.

GnRHa treatment also had a negative effect on RNA levels of the inhibin-subunit encoding genes, *INHA*, *INHBA*, and *INHBB*. Biologically active activins and inhibins are dimers composed of different α (encoded by *INHA*) and β subunits (βA encoded by *INHBA* and βB encoded by *INHBB*); the dimers include activin A (βAβA dimer), activin B (βBβB dimer), activin AB (βAβB dimer), inhibin A (αβA dimer), and inhibin B (αβB dimer). In the prepubertal testis, both *INHA* and *INHBB* subunits are expressed in Sertoli cells [7]. However, in pubertal and adult testis, *INHBB* is no longer expressed in Sertoli cells, but it continues to be expressed in germ cells and Leydig cells [7, 70, 71]. Serum inhibin B levels reflect Sertoli cell function [8, 37]. Serum inhibin B levels were reported to be normal in boys with cryptorchidism, including patients in the high infertility risk group (HIR/Ad-) [7, 21, 26]. During mini-puberty, inhibin B levels are elevated. Earlier studies of gonadotropin treatment in prepubertal boys with cryptorchidism showed that gonadotropin stimulation increased serum inhibin B levels in an age-dependent manner [9, 21]. In the present study, we did not evaluate inhibin B serum levels after GnRHa treatment, but the differential gene expression data of the single subunits (*INHA*, *INHBA*, and *INHBB*) pointed to gonadotropin-dependent transcriptional repression of both inhibin B and activin A. Importantly, upon treatment, although the expression of the protein encoding gene *INHBA* was repressed, the respective long non-coding RNA *INHBA-AS1* (logFC^GnRHa^ +2.02; [30]) was upregulated. Both *INHBA* and *INHBA-AS1* were previously associated with dental caries in genome-wide association studies [72], and *INHBA* was postulated to play a role in early tooth development [73]. Our results suggested that *INHBA-AS1* could be involved in the development of Sertoli cells. It was previously shown that activin A was involved, together with the germ-cell secreted protein, NODAL, in the upregulation of *NANOS2* during germ cell differentiation. Both activin A and NODAL bind activin A receptor type 2 (ACVR2A/2B) and type I receptors (ACVR1B/1C), but NODAL requires concomitant binding of the transmembrane co-receptor, CFC1/CRIPTO1, for activation. After GnRHa treatment, *ACVR2A* was found to be downregulated (logFC^GnRHa^ -0.60), and *ACVR1C* was upregulated (logFC^GnRHa^ +0.85). The overall negative effect of GnRHa, except for its effect on *ACVR1C*, suggested that the treatment-induced Ad spermatogonial development in infants was independent of the activation of inhibin B and the activin A pathway in Sertoli cells [26]. This finding was inconsistent with the hypothesis that inhibin B might be important for gonocyte transformation into Ad spermatogonia [25].

### FASLG, GDNF, and other genes with positive responses to GnRHa

*FASLG* expression was reported to be regulated by gonadotropin in human testis [74], and it was testosterone-dependent in rhesus monkeys [75]. With GnRHa treatment, we observed an increase in *FASLG* expression. This change could point to elevated apoptosis of Sertoli and germ cells; furthermore, it supported the notion that *FASLG* expression was dependent on testosterone and gonadotropin. Additionally, previous studies identified FASLG as target of matrix metallopeptidase 7 (MMP7), which proteolytically processes FASLG into a soluble form [76]. Interestingly, we also observed increases in *MMP7* RNA levels upon GnRHa treatment (logFC^GnRHa^ +2.52, FDR 0.0016), which suggested that soluble FASLG might be involved in GnRHa-induced germ cell development.

The influence of GDNF on the transition of gonocytes to spermatogonia in Ad- testes and the GnRHa dependence of *GDNF* expression was recently described [31]. GDNF was also reported to stimulate the proliferation of cultured, immature mouse Sertoli cells via its receptor subunit, NCAM, and the ERK1/2 signaling pathway [77]. Moreover, gonadotropin-dependent FSH stimulation was shown to induce increases in GDNF levels [78]; that interaction supported our findings that *GDNF* expression increased with GnRHa treatment. Furthermore, GDNF produced by peritubular myoid cells was reported to be testosterone-regulated and to be essential for repopulating the undifferentiated spermatogonial pool [79]. Therefore, a testosterone-dependent *GDNF* expression in Sertoli cells was likely, but a contribution of peritubular myoid *GDNF* RNA signaling to our data was also likely and has to be considered.

Sex hormone-binding globulin (SHBG/ABP), also known as androgen binding protein, is produced by Sertoli cells in most mammals. SHBG controls the availability of androgens in seminiferous tubules and in the epididymis (reviewed [80]). Transgenic mice that overexpressed rat SHBG developed progressive impairment of spermatogenesis [81]. Studies on human testis-specific SHBG revealed alternative splicing in exon 1. The Sertoli cell-specific SHBG transcripts included an exon 1 that lacked the secretion signal, and SHBG expression was repressed; the SHBG isoform with the other exon 1 was expressed in germ cells [82, 83]. Based on those findings, we excluded SHBG as a Sertoli cell specific gene in the present study. Nonetheless, we would like to point out that *SHBG* expression was found four fold increased after GnRHa treatment (logFC^GnRHa^ +2.00, FDR 0.0037). This finding supported the notion that *SHBG* expression was gonadotropin-dependent in the testis, which suggested that SHBG might be involved in the gonocyte transition to Ad spermatogonia.

### Limitations and outlook

To get an indication of differential expression of specific Sertoli cell expressing genes we selected for this study testicular RNA sequencing data of 40 Sertoli cell expressing genes whose expression profiles and protein products are commonly used as Sertoli cell specifiers. However, a contribution of RNA signals of other testicular cells in this study could not be excluded. Furthermore, changes at the mRNA level do not necessarily mean changes at the protein level. Nonetheless, differential RNA levels as determined by RNA profiling are a good indication that a given gene is implicated in a certain process. Therefore, it is convincing that, despite the above mentioned limitations, the observed differential expressions reflect the pathological state in Ad- testes and is the consequence of GnRHa treatment. In future, protein and gene expression of single cells should be tackled.

## Conclusion

This study showed that in Ad- testes with lower LH levels the expression of Sertoli cell specific genes, involved in cell morphology through complex gene networks, was affected positively and negatively. Based on these results, it is indicated that reduced LH and concomitantly reduced testosterone levels caused impaired Sertoli cell function and increased apoptosis.

GnRHa treatment mostly resulted in suppression of Sertoli cell specific genes. Based on our observations of differential gene expression of intermediate filaments and cell-cell junction proteins, we suggested that GnRHa triggered a restructuring process in Sertoli cells. Furthermore, we proposed that GnRHa induced *GDNF* and *FASLG* expression, which supported Sertoli cell proliferation and the gonocyte transition to Ad spermatogonia.

Finally, our results indicated that, during mini-puberty, LH and testosterone, but not inhibin B, were critical factors in driving the gonocyte transition to spermatogonia.

## Abbreviations

ACVR: Activin A receptor
AR: Androgen receptor
AMH: Anti-Mullerian hormone
BMP6: Bone morphogenetic protein 6
CALB2: Calbindin 2
CDKN1B: Cyclin-dependent kinase inhibitor 1B
CLDN11: Claudin 11
CLU: Clusterin
CREB1: cAMP response element binding protein 1
CTSL1: Cathepsin L
dbGaP: Database of Genotypes and Phenotypes
DES: Desmin
DMRT1: Doublesex and mab-3 related transcription factor 1
FDR: False Discovery Rate
FASLG: FAS ligand
FGF9: Fibroblast growth factor 9
FGFR2: Fibroblast growth factor receptor 2
FSH: Follicle stimulating hormone
FSHR: Follicle stimulating hormone receptor
GATA4: GATA Binding Protein 4
GDNF: Glial cell derived neurotrophic factor
GJA1: Gap junction protein alpha 1
GnRHa: Gonadotropin-releasing hormone agonist
HIR: High infertility risk
HPG: Hypothalamic-pituitary-gonadal
INHA: Inhibin alpha
INHBA: Inhibin beta A
INHBA-AS1: Inhibin beta A antisense RNA 1
INHBB: Inhibin beta B
KATNAL1: Katanin p60 subunit A-like 1
KITLG: KIT Ligand
KRT18: Keratin 18
LIR: Low infertility risk
LH: Luteinizing hormone
LogFC: Absolute fold change
MMP7: Matrix metallopeptidase 7
RHOX5: Reproductive homeobox 5
SERPINA5: Serpin peptidase inhibitor family A member 5
SOX8: SRY-Box 8
SOX9: SRY-Box 9
SSC: Spermatogonial stem cell
TJP1: Tight junction protein 1
VIM: Vimentin
WT1: Wilms tumor 1
ZFPM2: Zinc finger protein, FOG family member 2
